# Identification of a General *O-*linked Protein Glycosylation System in *Acinetobacter baumannii* and Its Role in Virulence and Biofilm Formation

**DOI:** 10.1371/journal.ppat.1002758

**Published:** 2012-06-07

**Authors:** Jeremy A. Iwashkiw, Andrea Seper, Brent S. Weber, Nichollas E. Scott, Evgeny Vinogradov, Chad Stratilo, Bela Reiz, Stuart J. Cordwell, Randy Whittal, Stefan Schild, Mario F. Feldman

**Affiliations:** 1 Alberta Glycomics Centre, Department of Biological Sciences, University of Alberta, Edmonton, Alberta, Canada; 2 Institut fuer Molekulare Biowissenschaften, Karl-Franzens-Universitaet Graz, Graz, Austria; 3 School of Molecular and Microbial Biosciences, University of Sydney, Sydney, New South Wales, Australia; 4 Institute for Biological Sciences, National Research Council, Ottawa, Ontario, Canada; 5 Defence Research and Development Canada Suffield, Medicine Hat, Alberta, Canada; 6 Alberta Glycomics Centre, Department of Chemistry, University of Alberta, Edmonton, Alberta, Canada; Tufts University School of Medicine, United States of America

## Abstract

*Acinetobacter baumannii* is an emerging cause of nosocomial infections. The isolation of strains resistant to multiple antibiotics is increasing at alarming rates. Although *A. baumannii* is considered as one of the more threatening “superbugs” for our healthcare system, little is known about the factors contributing to its pathogenesis. In this work we show that *A. baumannii* ATCC 17978 possesses an *O*-glycosylation system responsible for the glycosylation of multiple proteins. 2D-DIGE and mass spectrometry methods identified seven *A. baumannii* glycoproteins, of yet unknown function. The glycan structure was determined using a combination of MS and NMR techniques and consists of a branched pentasaccharide containing *N-*acetylgalactosamine, glucose, galactose, *N*-acetylglucosamine, and a derivative of glucuronic acid. A glycosylation deficient strain was generated by homologous recombination. This strain did not show any growth defects, but exhibited a severely diminished capacity to generate biofilms. Disruption of the glycosylation machinery also resulted in reduced virulence in two infection models, the amoebae *Dictyostelium discoideum* and the larvae of the insect *Galleria mellonella*, and reduced *in vivo* fitness in a mouse model of peritoneal sepsis. Despite *A. baumannii* genome plasticity, the *O*-glycosylation machinery appears to be present in all clinical isolates tested as well as in all of the genomes sequenced. This suggests the existence of a strong evolutionary pressure to retain this system. These results together indicate that *O*-glycosylation in *A. baumannii* is required for full virulence and therefore represents a novel target for the development of new antibiotics.

## Author Summary

Multidrug resistant (MDR) *Acinetobacter baumannii* strains are an increasing cause of nosocomial infections worldwide. Due to the remarkable ability of *A. baumannii* to gain resistance to antibiotics, this bacterium is now considered to be a “superbug”. *A. baumannii* strains resistant to all clinically relevant antibiotics known have also been isolated. Although MDR *A. baumannii* continues to disseminate globally, very little is known about its pathogenesis mechanisms. Our experiments revealed that *A. baumannii* ATCC 17978 has a functional *O*-linked protein glycosylation system, which seems to be present in all strains of *A. baumannii* sequenced to date and several clinical isolates. We identified seven glycoproteins and elucidated the structure of the glycan moiety. A glycosylation-deficient strain was generated. This strain produced severely reduced biofilms, and exhibited attenuated virulence in amoeba, insect, and murine models. These experiments suggest that glycosylation may play an important role in virulence and may lay the foundation for new drug discovery strategies that could stop the dissemination of this emerging human pathogen, which has become a major threat for healthcare systems.

## Introduction

*Acinetobacter baumannii* is a strictly aerobic Gram negative, non-fermentative, opportunistic pathogen. Since the 1970's, this organism has frequently been isolated from healthcare facilities, but was easily controlled with antibiotics [1], [2]. However, many clinical isolates of *A. baumannii* have recently emerged with extreme resistance to antibiotics, disinfectants, and desiccation, which has permitted *A. baumannii* to disseminate throughout healthcare facilities worldwide [3]–[7]. One recent study showed that from 2001 to 2008 the percentage of *A. baumannii* isolates resistant to at least three classes of antibiotics increased from 4% to 55%, and 17% of all isolates were resistant to at least four drug classes [8]. Panresistant strains of *A. baumannii* have also been isolated [9]. Because of its importance as an emerging pathogen, attention towards *A. baumannii* has increased considerably. Most of the efforts have focused on antibiotic resistance mechanisms, but little is known about its virulence factors. A significant amount of work has been done to characterize biofilm formation, which seems to play a role in pathogenesis [10], [11]. Other suggested virulence factors for *A. baumannii* include the capsule, exopolysaccharide, pili and lipopolysaccharide (LPS) [11]–[14]. Undoubtedly, more research is needed in order to understand *A. baumannii* pathogenesis.

Genomic analysis of all sequenced *A. baumannii* strains revealed the presence of homologous genes to those encoding enzymes involved in the *Neisseria meningitidis* protein *O-*glycosylation system. Many different mucosal pathogenic bacteria require protein glycosylation for virulence, and glycoproteins seem to play a role in adhesion, motility, DNA uptake, protein stability, immune evasion, and animal colonization [15]. Whereas *N*-glycosylation seems to be restricted to epsilon and a few delta proteobacteria, *O*-glycosylation appears to be more widespread among bacteria. Gram negative bacteria including *Neisseria spp.* and *Bacteroides fragilis* employ *en bloc O*-glycosylation as a general system to modify multiple proteins [16], [17]. *En bloc O-*glycosylation is initiated by a specialized glycosyltransferase that attaches a nucleotide-activated monosaccharide-1P to an undecaprenolphosphate (Und-P) lipid carrier on the inner face of the inner membrane. A series of glycosyltransferases subsequently attach additional monosaccharides to the first sugar residue on Und-PP. When the carbohydrate structure is completed, the Und-PP linked glycan is flipped to the periplasmic face, where an *O*-oligosaccharyltransferase (*O-*OTase) transfers the carbohydrate to selected Ser or Thr residues in acceptor proteins [18], [19]. *Campylobacter jejuni* employs a similar *N-*glycosylation pathway to modify about 65 proteins [20].

This work demonstrates the existence of a general *O-*glycosylation system in *A. baumannii* ATCC 17978, which is required for efficient biofilm formation and pathogenesis in the *Dictyostelium discoideum*, *Galleria mellonella*, and murine septicemia virulence models. We identified seven glycoproteins carrying a branched pentasaccharide, the structure of which has been characterized by MS and NMR techniques. *O-*glycosylation appears to be ubiquitous in *A. baumannii*, which suggests that this system might be a possible target for novel antimicrobial treatments.

## Results

### Identification of an *O-*OTase homologue in *A. baumannii* and construction of an in-frame knockout mutant

We initially searched the *A. baumannii* ATCC 17978 genome for homologues of known *O-*OTases. Via a BLAST analysis, we identified a homolog to the *N. meningitidis O-*OTase PglL (A1S_3176; E-value 1e-9) that contained a Wzy_C motif. This motif is conserved in all *O*-OTases, but is also found in WaaL ligases, which catalyze the transfer of the O antigen to the Lipid A core [21], [22]. To date, only experimental determination allows the assignment of an ORF containing the Wzy_C motif as either an *O*-OTase or a ligase [23]. No other ORFs contained a Wzy_C motif in the *A. baumannii* ATCC 17978 genome. A1S_3176 is not predicted to be part of an operon [24]. We carried out mutagenesis of the A1S_3176 gene by homologous recombination to evaluate if its encoded protein is an *O*-Otase or a WaaL ligase. PCR and DNA sequencing confirmed the creation of an A1S_3176 knockout strain, in which the targeted gene was replaced with a gentamicin resistance cassette. There was no significant difference between the growth curves of the wild-type and the A1S_3176 mutant strains at 37°C, indicating that growth in these conditions was not affected by the mutation (Data not shown).

### A1S_3176 (PglL_Ab_) is required for glycosylation of membrane proteins in *A. baumannii* ATCC 17978

**Figure 1:**
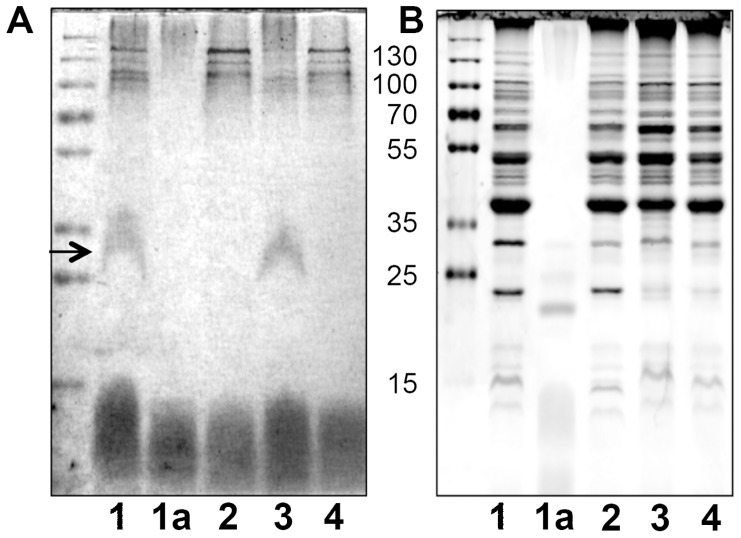
*A. baumannii* requires PglL_Ab_ to glycosylate membrane proteins. 10 µg of membrane extract from *A. baumannii* strains (lanes 1,2,3,4) were resolved by SDS-PAGE. A) Carbohydrates were detected by PAS stain. B) Proteins were detected by Coomassie staining. Samples were as follows: lane 1 WT; lane 1a Proteinase K treated WT; lane 2 Δ*pglL*, lane 3 Δ*pglL*, pWH1266-*pglL*, 4-Δ*pglL*, pWH1266 control.

Most of the *Neisseria O*-glycoproteins identified to date are associated to membranes [16]. Membrane extracts from wild type and ΔA1S_3176 *A. baumannii* strains were analyzed by SDS-PAGE followed by PAS staining, a technique that is specific for detecting glycans, but presents low sensitivity (Fig. 1). A broad band migrating from 25 to 35 kDa was visualized in the extract of *A. baumannii* WT. Although the membrane protein profile between the WT and the ΔA1S_3176 strains appeared similar, the band detected via PAS stain was not visible in the mutant strain, suggesting that A1S_3176 is required for glycosylation of at least one protein (Fig. 1B). The PAS-reactive band disappeared upon treatment with proteinase K, associating the glycan signal with proteinaceous material. Complementation of A1S_3176 was achieved *in trans*, and analysis of *A. baumannii* ΔA1S_3176-pWH1266-*pglL* membrane extract showed the reappearance of the PAS stained band. Due to the aforementioned similarity between *O-*OTases and ligases, we carried out a conventional LPS extraction and analyzed the extract of the different strains via SDS-PAGE. Silver stain showed no obvious differences in the carbohydrate pattern were observed, suggesting that A1S_3176 is not involved in LPS synthesis (Fig S1). To further determine if A1S_3716 effected LPS biosynthesis, whole cells were digested with proteinase K and analyzed by Silver stain and no differences were observed (data not shown). However, it has been reported that the O-antigen chains of certain *A. baumannii* strains are not detectable by Silver stain and therefore we cannot conclusively exclude a role of A1S_3176 in LPS synthesis [25]. Together these results suggest that A1S_3176 is an *O-*OTase responsible for *O*-glycosylation in *A. baumannii* and will be referred from here on as PglL_Ab_, as per its *N. meningitidis* ortholog.

### Identification of two glycoproteins in *A. baumannii* via 2D-DIGE and preliminary characterization of the *O-*glycan by MALDI-TOF/TOF MS and MS/MS analysis

**Figure 2:**
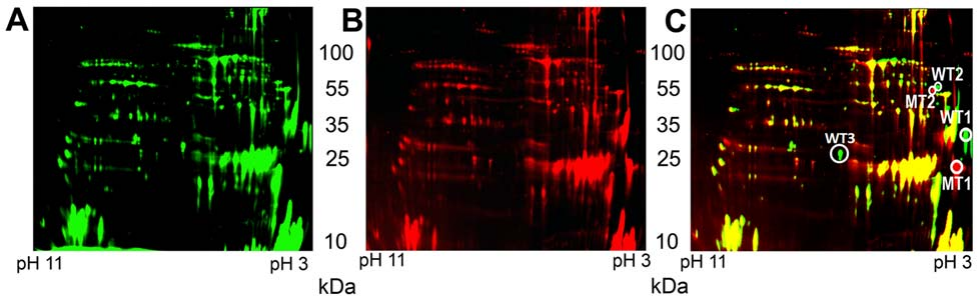
Comparison of *A. baumannii* WT and Δ*pglL* membrane extracts by 2D-DIGE. Analysis of the membrane proteome of *A. baumannii* WT strain (A), Δ*pglL* strain (B), and merge (C). Spots WT1 and WT2 only present in the WT strain (green) whereas MT1 and MT2 were only present in the Δ*pglL* strain (red). MALDI-TOF MS analysis identified WT1 and MT1 spots as A1S_3626 protein and WT2 and MT2 spots as A1S_3744 protein.

To identify the glycoprotein(s) in *A. baumannii*, we performed two dimensional in-gel electrophoresis (2D-DIGE) experiments [26]. Membrane samples of both WT and Δ*pglL* were isolated by ultracentrifugation and the lipidic components were removed as previously described [27]. Most of the signals corresponding to the wild type (Fig. 2A, green) and Δ*pglL* (Fig. 2B, red) proteins co-localized in the gel (Fig. 2C, yellow), indicating that these proteins were likely not glycosylated. However, a few proteins exhibited differential electrophoretic behavior (Fig. 2). These proteins spots were excised, in-gel digested, and analyzed by MALDI-TOF/TOF MS and MS/MS. We identified two separate pairs of proteins, which according to their electrophoretic migration, appeared to be larger and more acidic in the WT strain (WT1 and WT2) than in the Δ*pglL* strain (MT1 and MT2). Mass spectrometric analysis determined WT1 and MT1 samples to be A1S_3626 protein, whereas WT2 and MT2 were identified as A1S_3744 protein. Both, A1S_3626 and A1S_3744 are annotated as hypothetical proteins, and BLAST searches yielded homologues exclusively within the *Acinetobacter* genus.

**Figure 3:**
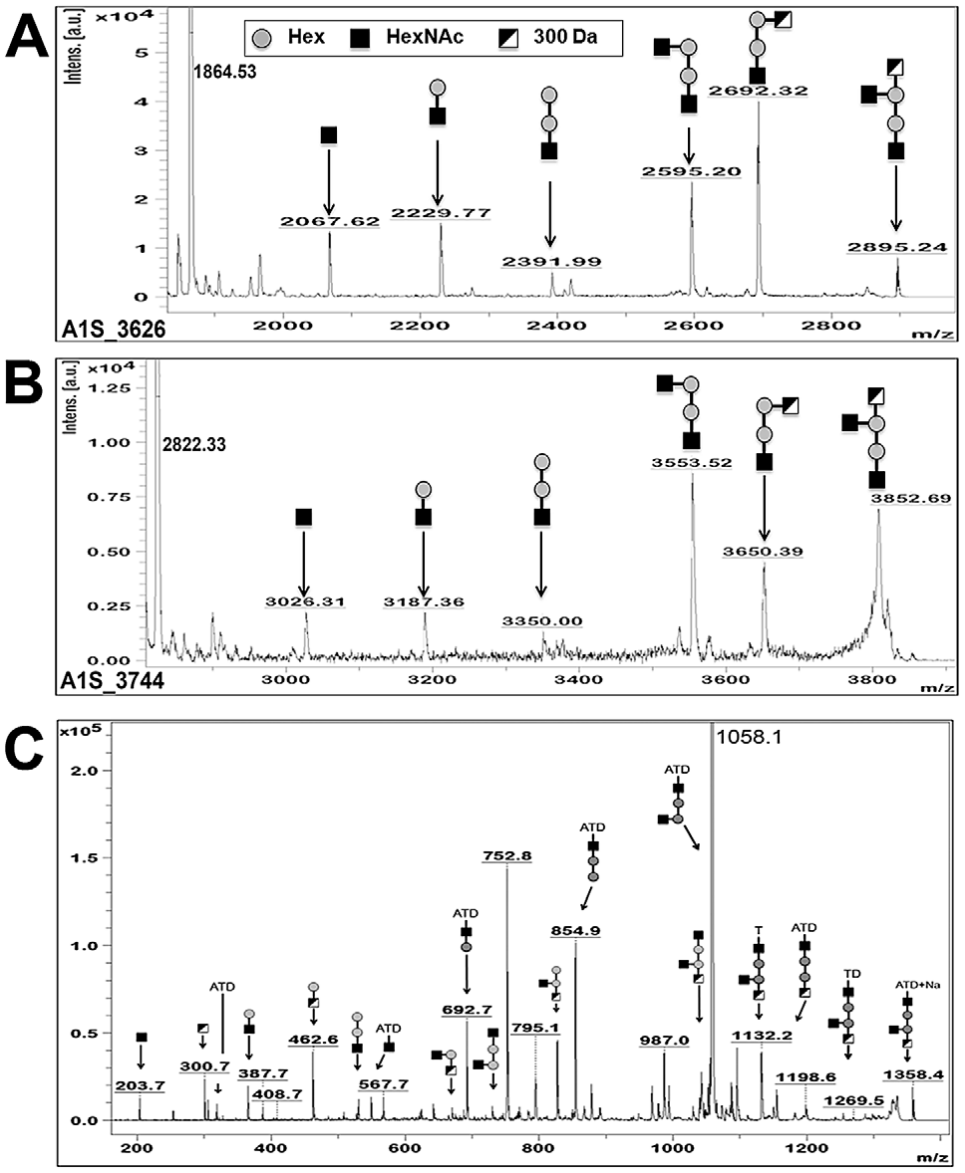
MS/MS of A1S_3626 and A1S_3744 showing glycosylation in *A. baumannii* with a pentasaccharide. Spots excised from the 2D DIGE, digested with trypsin, and analyzed by MALDI-TOF-MS. Peaks not corresponding to peptide fragmentation were analyzed for glycosylation. A) MS/MS of the precursor ion peak at *m/z* 2895.165 from A1S_3626 revealed the peptide **SAGDQAASDIATATDNASAK** with a pentasaccharide of HexNAc-Hex-Hex-(HexNAc)-300 attached. B) MS/MS of the precursor ion peak at *m/z* 3852.76 from A1S_3744 revealed the peptide **ETPKEEEQDKVETAVSEPQPQKPAK** with the same pentasaccharide attached. C) MALDI-TOF MS of Pronase E digested membrane proteins showed a precursor ion peak of *m/z* 1358.4 which MS/MS analysis demonstrated to be the previously identified *O*-glycan (HexNAc-Hex-Hex-(HexNAc)-300 attached to the peptide fragment “ATD”.

Analysis of the MALDI-TOF MS spectra of a tryptic digest of WT1 (A1S_3626) revealed a peptide fragment of 2895.24 Da that was absent in MT1 (Fig. 3A). MALDI-TOF-TOF MS/MS of this ion determined that in the wild-type strain the peptide SAGDQAASDIATATDNASAK was linked to the glycan HexNAc-Hex-Hex-(HexNAc)-300, where 300 corresponded to an unknown residue of *m/z* 300, whereas the same peptide was unmodified in *ΔpglL* sample (Fig S3A). Similarly, MALDI-TOF MS analysis of a tryptic digest of WT2 (A1S_3744) revealed a peptide fragment of 3852.69 Da that was absent in MT2 (Fig. 3B). MALDI-TOF-TOF MS/MS of the 3852.69 Da peak revealed the same pentasaccharide identified on A1S_3626 on the peptide ETPKEEEQDKVETAVSEPQPQKPAK (2822.33 Da), whereas the same peptide was unmodified in *ΔpglL* sample (Fig S3B).. We next purified membranes from *A. baumannii*, digested the sample with Pronase E, and enriched glycosylated peptides using activated charcoal microspin columns. We identified a peak in the MALDI-TOF MS of 1358.4 m/z that was subsequently analyzed by MALDI-TOF/TOF MS/MS (Fig. 3C). Manual peak annotation identified the previously characterized pentasaccharide attached to a sodiated tripeptide containing the amino acids A, T and D. Overall, these results demonstrate that **PglL** ***_Ab_*** **glycosylates at least two different proteins with a pentasaccharide** with a preliminary structure of HexNAc-Hex-Hex-(HexNAc)-300.

We observed other spots possibly corresponding to proteins migrating differently in *A. baumannii* WT and *ΔpglL* strains. The most prominent was marked as WT3, and was observed only in the WT extract (Fig. 2C). Mass spectroscopy analysis determined this spot corresponded to OmpA (A1S_2840). However, manual analysis using MS/MS of WT3 indicated that OmpA was not glycosylated. Western blot analysis of whole cell extracts of the WT and *ΔpglL* strains revealed no difference in OmpA expression levels, which implies that manipulation of membrane samples could account for apparent differences observed in expression levels of proteins detected by 2D-DIGE (Fig S2).

### Identification of additional *O-*glycosylated proteins in *A. baumannii* ATCC 17978 by zwitterionic hydrophilic interaction chromatography (ZIC-HILIC) MS/MS

**Figure 4:**
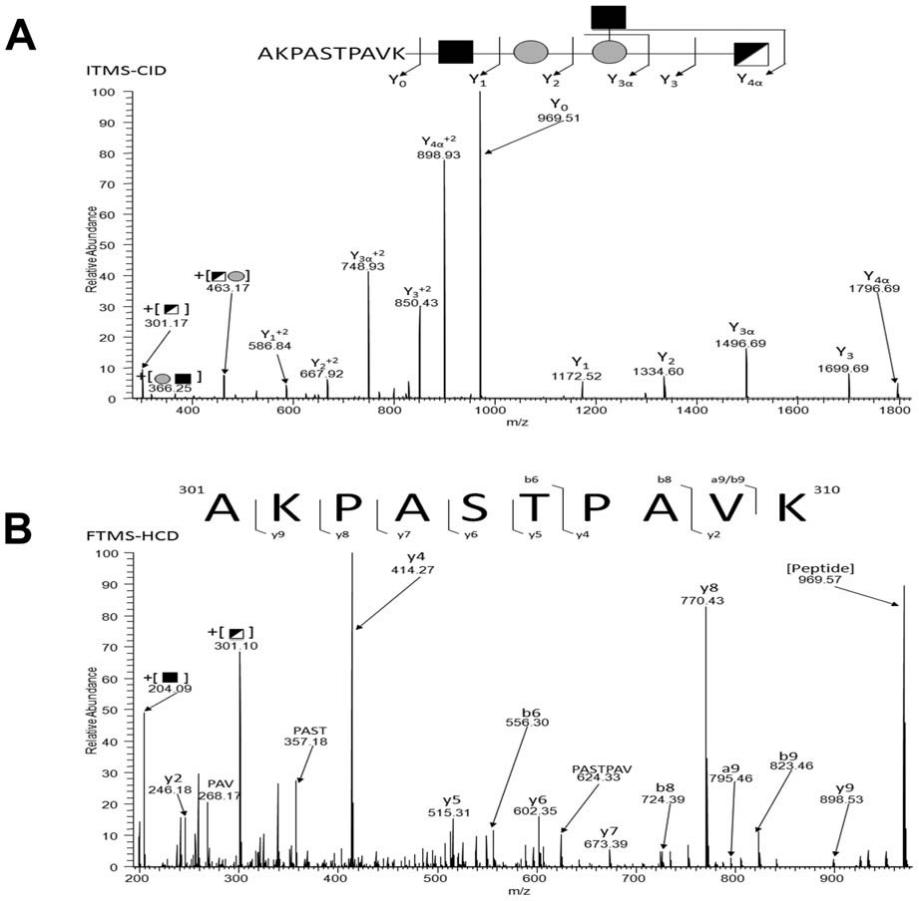
Identification of additional glycoproteins in *A. baumannii* ATCC 17978. Tryptically digested membranes were enriched via ZIC-HILIC and analyzed by LC-MS and HCD MS-MS. All spectra were analyzed for the diagnostic oxonium ion of 301.10 m/z, and positive spectra were analyzed manually to identify the glycopeptide. This spectra is representative of each glycopeptide identified in Table 2. A) ITMS-CID of the precursor ion at *m/z* 1999.943 reveals the pentasaccharide attached to the peptide **AKPASTPAVK**. B) FTMS-HCD of the precursor ion at *m/z* 1999.943 reveals the peptide sequence **AKPASTPAVK**.

To determine if additional glycoproteins were present in *A. baumannii* ATCC 17978, we employed ZIC-HILIC glycopeptide enrichment. Utilizing membrane extracts previously shown to contain A1S_3626 and A1S_3744 putative glycopeptides were enriched and analyzed using an LTQ-Orbitrap Velos. HCD scans containing oxonium ion were manually inspected and searched using MASCOT resulting in the identification of at least 9 different glycosylation sites on 7 different glycoproteins in *A. baumannii* ATCC 17978 (Table 1; Fig. 4). This peptide-centric approach enabled multiple novel glycoproteins to be identified of which six of the seven proteins are annotated as uncharacterized hypothetical proteins, with the remaining being annotated as MotB (A1S_1193). (Table 1). This demonstrates that PglL_Ab_ is able to glycosylate multiple proteins in *A. baumannii* ATCC 17978.

**Table 1:** Identification of seven *O-*glycosylated proteins with nine unique glycosylation sites by ZIC HILIC enrichment of tryptically digested *A. baumannii* ATCC 17978 membrane extracts.

Precursor m/z [Da]	MH+ [Da]	Charge	Peptide mass [Da]	Mascot Ion score	Sequence	protein name	Annotation for *A. baumannii* ATCC 17978
749.02	2245.040	3	1214.68	31	^301^AKPASTPAVKSAS^313^	Putative uncharacterized protein	A1S_0556
1000.47	1999.943	2	969.57	49	^301^AKPASTPAVK^310^	Putative uncharacterized protein	A1S_0556
999.95	3996.794	4	2966.41	40	^108^AAQADKKTEASAAATTEQQDSFDAQVQR^135^	Putative uncharacterized protein	A1S_0556
1090.53	5448.631	5	4418.21	39	^203^AASGVEAAAAPATLTLSTDDKGAVSQCQAGIGDQGFLATLQTQVK^247^	OmpA/MotB	A1S_1193
1144.20	3430.587	3	2400.20	81	^78^NLQKAEDQNADSGIAASTPVATAK^101^	Putative uncharacterized protein	A1S_2371
1344.64	4031.899	3	3001.51	86	^72^ASTTLQNLQKAEDQNADSGIAASTPVATAK^101^	Putative uncharacterized protein	A1S_2371
1469.05	4405.132	3	3374.73	51	^22^ASTTEQPLNPNKVSAPVEDPIDPLAVDAASTVK^54^	Putative uncharacterized protein	A1S_2371
1210.52	2420.037	2	1389.66	53	^52^SDAVGSASEAAPATR^66^	Putative uncharacterized protein	A1S_3580
1281.56	2562.115	2	1531.73	74	^50^AASDAVGSASEAAPATR^66^	Putative uncharacterized protein	A1S_3580
1283.06	2565.111	2	1534.73	47	^52^QAASDIATATDNASAK^67^	Putative uncharacterized protein	A1S_3626
1406.62	4217.856	3	3187.46	77	^48^SAGDQAASDIATATDN(+1)ASAKIDAAADHAADATAK^81^	Putative uncharacterized protein	A1S_3626
965.75	2895.252	3	1864.85	51	^48^SAGDQAASDIATATDNASAK^67^	Putative uncharacterized protein	A1S_3626
1054.98	4216.890	4	3186.48	66	^48^SAGDQAASDIATATDNASAKIDAAADHAADATAK^81^	Putative uncharacterized protein	A1S_3626
755.54	3019.146	4	1988.77	24	^28^NDGM(+16)HEASDPATSHDM(+16)NK^45^	Putative uncharacterized protein	A1S_3658
996.39	2987.158	3	1956.78	80	^28^NDGMHEASDPATSHDMNK^45^	Putative uncharacterized protein	A1S_3658
797.81	3188.233	4	2157.86	22	^28^NDGMHEASDPATSHDMNKNS^47^	Putative uncharacterized protein	A1S_3658
1001.72	3003.153	3	1972.78	42	^28^NDGM(+16)HEASDPATSHDMNK^45^	Putative uncharacterized protein	A1S_3658
1133.18	3397.538	3	2367.17	44	^29^EEEQDKVETAVSEPQPQKPAK^49^	Putative uncharacterized protein	A1S_3744
880.42	2639.232	3	1608.86	55	^35^VETAVSEPQPQKPAK^49^	Putative uncharacterized protein	A1S_3744

### Structure determination of the *O-*linked glycan by 2D NMR

Identification of the *O-*glycan of *A. baumannii* ATCC 17978 was achieved by 2D NMR analysis. The Pronase E digested membrane protein extracts characterized in Fig. 3C were analyzed by ^1^H:^13^C HSQC 2D NMR and revealed the structure of the pentasaccharide to be β-GlcNAc3NAcA4OAc-4-(β-GlcNAc-6-)-α-Gal-6-β-Glc-3-β-GalNAc-, with the amino acids S, E, and A attached in any combination (Fig S4, Table 2). β-GlcNAc3NAcA4OAc (corresponding to m/z 300; Fig. 3) is an *O*-acetylated derivative of glucuronic acid, and can account for the more acidic migration of the WT glycoproteins compared to the *ΔpglL* in the 2D-DIGE analysis.

**Table 2:** ^1^H∶^13^C HSQC 2D NMR data for the characterization of the *A. baumannii* 17978 *O-*glycan.

Unit		H/C-1	H/C-2	H/C-3	H/C-4	H/C-5	H/C-6a;b
Gal A	H	4.95	3.68	3.94	4.12	4.02	3.78; 4.12
	C	99.3	69.8	70.6	79.1	70.8	72.3
GlcNNA B	H	4.84	3.92	4.20	4.96	3.99	
	C	103.4	54.5	53.9	71.9	76.0	174.4
GlcNNA B′	H	4.81	3.79	3.99	3.64	3.88	
	C	103.5	55.0	56.1	71.1		
GalNAc C	H	4.58	4.07	3.87	4.14	3.70	3.77; 3.80
	C	101.9	52.0	81.4	69.0	76.1	62.2
Glc D	H	4.53	3.30	3.47	3.50	3.61	3.69; 3.88
	C	105.7	74.0	76.9	70.6	75.5	66.8
GlcNAc E	H	4.50	3.70	3.52	3.45	3.45	3.77; 3.94
	C	103.1	56.7	75.2	71.0	77.0	61.8
Ser	H		4.30	4.14; 4.14			
	C	178.2	54.3	68.1			
Glu	H		4.45	2.02; 2.14	2.51; 2.51		
	C	178.4	54.2	27.7	31.4		
Ala	H		4.42	1.42			
	C	175.9	51.0	17.7			
Ala*	H		4.31	1.42			
	C	178.4	51.0	17.7			

*: Indicates impurity.

### PglL_Ab_ is required for efficient biofilm formation

**Figure 5:**
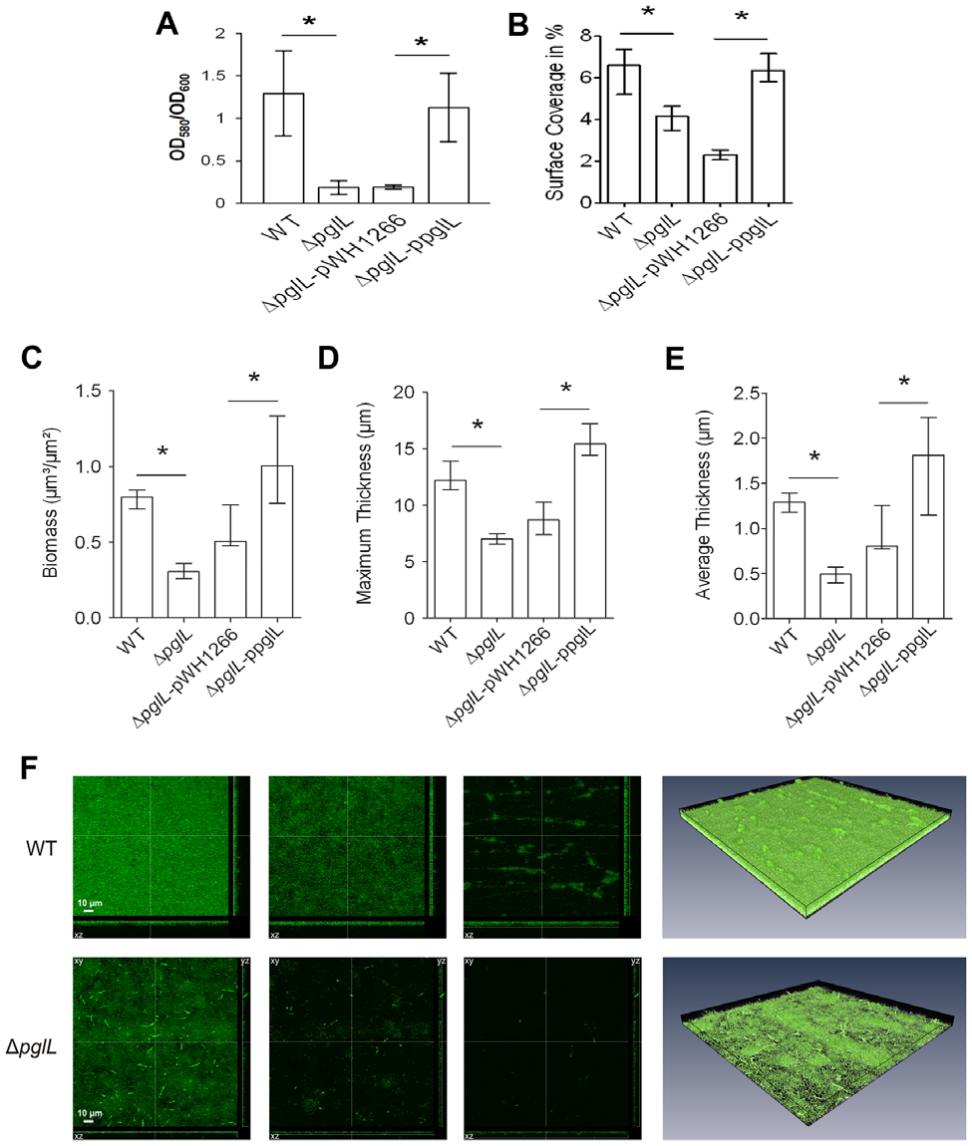
*A. baumannii* requires PglL_Ab_ for biofilm formation. A) Quantitative biofilm formation on polystyrene 96 well plates by strains incubated without perturbation in LB at 30°C. The bars indicate the means for 8 replicates. The error bars indicate the standard deviation of the means. Asterisks indicate significant differences (*, *P*<0.005 [*t* test; *n* = 8]; **, *P*<0.001 [*t* test; *n* = 8]). B) The median surface coverage after incubation for 2 h in flow cell chambers of the WT, *ΔpglL*, *ΔpglL* pWH1266 and *ΔpglL* ppglL was determined by the COMSTAT software. For each strain at least six micrographs from three independent experiments were analyzed. The error bars indicate the interquartile range. Asterisks indicate significant differences (*, *P*<0.05 [Mann-Whitney U test; *n* = 6]). C)–E) Image stacks of the WT, *ΔpglL*, *ΔpglL* pWH1266 and *ΔpglL* ppglL biofilms grown in flow cells for 24 h were analyzed for the biomass as well as the maximum and average thickness using the COMSTAT software. Shown are the medians of at least six image stacks from three independent experiments for each strain. The error bars indicate the interquartile range. Asterisks indicate significant differences (*, *P*<0.05 [Mann-Whitney U test; *n* = 6]). F) Shown are representative confocal laser scanning microscopy images of the WT (upper row) and *ΔpglL* mutant (lower row) biofilms grown in flow cells for 24 h. The first three images represent horizontal (xy, large panel) and vertical (xz and yz, side panels) projections at different z-levels (from left to right 0.2 µm, 3 µm and 6 µm). The fourth micrograph of each row represents a three-dimensional image analyzed by the AMIRA software package of the WT and *ΔpglL* mutant biofilms, respectively.

It has been suggested that biofilm formation is important for *A. baumannii* virulence [28]. We tested if *O-*glycosylation has an impact on biofilm formation in this organism. Biofilm formation was detected using crystal violet staining and quantitatively analyzed by comparing the ratio between cell growth (OD_600_) and biofilm formation (OD_580_) at 30°C after 48 hours incubation (Fig. 5A). High absorbance values corresponding to a strong ability to create biofilms (1.23±0.48 and 1.12±0.40) were obtained for the WT strain and the Δ*pglL* strain complemented *in trans* respectively. On the contrary, the Δ*pglL* strain and the Δ*pglL* strain transformed with pWH1266 exhibited severely reduced levels of absorbance (0.18±0.07 and 0.20±0.04). Similar results were also observed at 37°C (data not shown). We further characterized the role of *O-*glycosylation in biofilm formation by employing a flow cell system. *A. baumannii* strains were stained with the green fluorescent stain SYTO 9, visualized by confocal laser scanning microscopy, and quantitative analysis of the biofilms was performed with COMSTAT. Assessment of the initial attachment after 2 hours shows that Δ*pglL* strain and vector control had significantly less surface coverage (4.12% and 2.32% respectively) than the WT and *in trans* complemented strain (6.41% and 6.45% respectively; Fig. 5B). Confocal microscopy and subsequent analysis of biofilms biomass, as well as average and maximal thickness after 24 hours showed significantly higher levels for the WT compared to the Δ*pglL* strain, and the phenotype was restored to WT levels when *pglL_Ab_* was complemented *in trans* (Fig. 5 C, D, E, F; *P<0.05). These data indicate that the *A. baumannii* strain defective in *O*-glycosylation has a severely diminished capacity to form biofilms.

### PglL_Ab_ is required for virulence towards *Dictyostelium discoideum*, and *Galleria mellonella*

**Figure 6:**
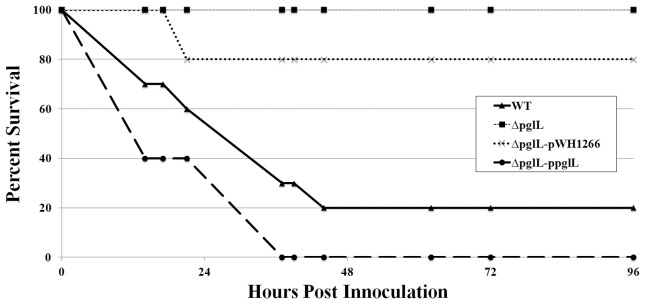
*A. baumannii* pathogenesis is dependent on PglL_Ab_ in the *Galleria mellonella* virulence model. Representative data of survival rate of 3 biological replicates of 10 individual *G. mellonella* injected with 2.31±1.13×10^5^ CFU of each strain in 5 µL of sterilized PBS and incubated @ 37°C. Survival was assayed by response to touch or discoloration. While killing by WT and *ΔpglL-*pglL was observed, no killing was observed in the Δ*pglL* and 20% killing was observed in the *ΔpglL*-pWH1266 strains up to 96 hours. No killing was observed in the PBS injection control for the length of the experiment.

Two well-established virulence models for *A. baumannii* are the *D. discoideum* predation and the *G. mellonella* infection models [5], [29]–[33]. *D. discoideum* is an unicellular amoeba that feeds on bacteria and previous work has demonstrated similarity between phagocytosis of the amoebae and mammalian phagocytes [34]. We examined if protein glycosylation was required for virulence towards *D. discoideum* by co-incubation of *A. baumannii* strains with the amoebae on SM/5 nutrient agar. *A. baumannii* was previously shown to inhibit amoebae growth in the presence of 1% ethanol [5]. The WT strain was virulent and inhibited all *D. discoideum* growth in the presence of 1% ethanol, which resulted in no plaque being formed. However the Δ*pglL* strain was avirulent towards the amoeba, which resulted in plaque formation in the bacterial lawn within 48 hours and clearing of the plate within 4–5 days (Fig S5). *G. mellonella* have been used to study many host-pathogen interactions, and have several advantages over other virulence models including the presence of both humoral (ie. antimicrobial peptides) and cellular immune response systems (phagocytic cells) [32]. Most importantly, a correlation has been established between the virulence of several bacteria in *G. mellonella* and mammalian models [35], [36]. For the *G. mellonella*, while a similar bacterial load (2.31±1.13×10^5^ CFU) was injected for each of the strains, only the WT and complemented strains were able to kill the wax moth larvae after 36 hours, (20% and 0% survival), whereas larvae injected with Δ*pglL* and the Δ*pglL* vector control strains had significantly higher survival rates (100% and 80%; Fig. 6). The LD_50_ of the WT and complemented strains were determined to be approximately 2.6×10^4^ and 1.4×10^4^ respectively after 36 hours. No additional killing was observed in the *ΔpglL* or vector control strains up to 96 hours. A PBS injected control maintained 100% survival throughout the length of the virulence assay. These results demonstrate a critical role for *O*-glycosylation in the virulence of *A. baumannii* in these two model systems.

### PglL_Ab_ is required for competitive fitness in BALB/c mice

**Figure 7:**
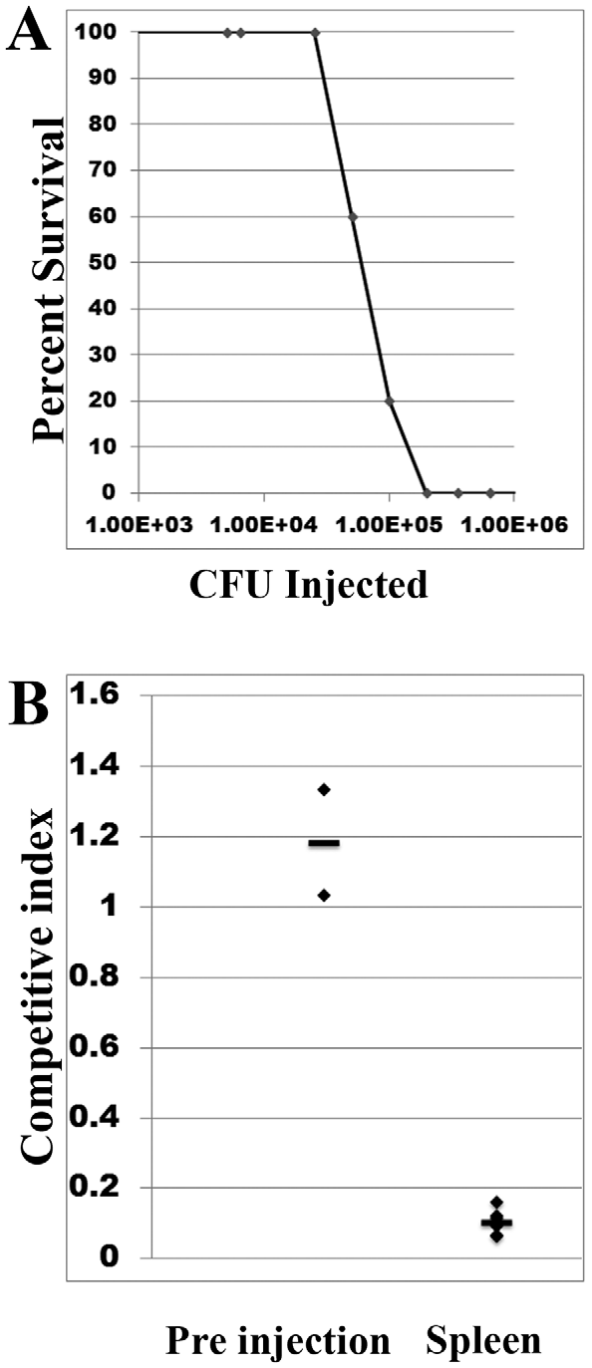
Characterization of *A. baumannii* ATCC 17978 pathogenesis in a murine septicemia model. A) Determination of the LD_50_ of *A. baumannii* ATCC 17978. Groups of 5 mice were injected with serial dilutions of *A. baumannii* WT to determine the LD_50_ which was calculated to be 6.49×10^4^ CFU @ 18 hrs post infection. B) Murine competition septicemia between *A. baumannii* WT and *ΔpglL*. Groups of 3 mice were injected with ∼1∶1 WT to Δ*pglL* CFU's and were sacrificed after 18 hrs, spleens were harvested, and bacterial load determined.

We then tested *A. baumannii ΔpglL* virulence *in vivo* using a previously described murine septicemia competition model [37]–[39]. We first determined the LD_50_ of *A. baumannii* ATCC 17978 strain by injecting groups of 5 BALB/c mice with serially diluted bacteria cultures (Fig. 7A). A very small dose range between full survival and full killing was observed, and the LD_50_ was determined to be 6.49×10^4^ CFU/mouse. The competition index (CI) was defined as the number of *ΔpglL* CFUs recovered/number of WT CFUs recovered, divided by the number of *ΔpglL* CFUs inoculated/number of WT CFUs inoculated. Cultures of each strain were mixed at a ratio of 1∶1, serial diluted, and plated to determine the initial CI. 1×10^5^ CFU of the mixed strains were injected intraperitoneally into the BALB/c mice, which were subsequently sacrificed 18 hrs post injection. The spleens were aseptically harvested, serial diluted, and plated. All of the mice had a high spleen CFU load of 3.75±2.37×10^8^ CFU/gram and were moribund at the time of sacrifice. While the initial prescreen showed a CI of 1.18±0.21 favoring the *ΔpglL* mutant, the spleen counts after 18 hrs showed a CI of 0.10±0.03 (Fig. 7B). This data suggests that Ab *ΔpglL* has a competitive disadvantage as compared to the WT strain. Together, these results indicate that *A. baumannii* strains lacking *O*-glycosylation are attenuated in mice.

### Protein glycosylation appears to be ubiquitous in *Acinetobacter* sp

To determine the degree of conservation of the *O-*glycosylation system in *Acinetobacter* sp., we searched for the presence of PglL_Ab_ homologues in different species within the genus. This genomic search showed that PglL*_Ab_* was present in all the genomes analyzed with high sequence homology (Fig S6A). We obtained eight clinical isolates from the University of Alberta Hospital. The isolates were identified by 16S rDNA and *recA* sequencing to be different species within the *Acinetobacter* genus (*A. baumannii*, *A. nosocomialis*, *A. pittii*, and *A. calcoaceticus*). Membranes of these strains were purified and analyzed by PAS staining for the presence of glycoproteins (Fig S6B). While there appears to be variation in the size and intensity of the PAS stained band, all the isolates were positive for glycoproteins, demonstrating that PglL_Ab_ was active in all these strains. This indicates that despite the plasticity of *Acinetobacter* sp. genomes [40], there is a strong evolutionary pressure to retain a functional *O-*glycosylation system.

## Discussion

Isolation of MDR strains of *A. baumannii* is increasing at impressive rates. Despite its growing incidence as nosocomial pathogen, only a few *A. baumannii* virulence factors have been characterized. In this article we describe a general *O*-glycosylation system in *A. baumannii* ATCC 17978. Although once considered rare in prokaryotes, both *N*- and *O*-glycoproteins are present in all domains of life. In most bacterial species known to synthesize glycoproteins, glycosylation is restricted to a few proteins including adhesins, flagellins or pilins [15]. Only a few “general” glycosylation systems in which more than a single protein is glycosylated have been characterized. *C. jejuni N*-glycosylates more than 65 proteins with the same heptasaccharide. Inactivation of the glycosylation pathway does not have an effect on growth *in vitro*, but does reduce adhesion and invasion to cells in culture, and affects chicken and mice colonization [41]. *Neisseria gonorrhoeae* is able to *O*-glycosylate at least 12 proteins with a highly variable glycan structure [42]. The glycan has recently been shown to be important for infection of cervical epithelial cells [43]. *Bacteroides fragilis* also has a general *O*-glycosylation system, where hundreds of proteins are predicted to be glycosylated [44]. Inactivation of the glycosylation system results in severe growth defects *in vitro* [17]. It was then not surprising to see that the glycosylation mutant strain was outcompeted by the wild-type strain in gnotobiotic mice colonization experiments. Seven proteins are shown to be *O*-glycosylated by the PglL OTase encoded by the A1S_3176 gene. Cells unable to perform protein glycosylation do not show any differential growth phenotype *in vitro*, while exhibiting a diminished capacity to form biofilms and reduced virulence in *D. discoideum*, *G. mellonella*, and murine septicemia pathogenesis models systems.

Two glycoproteins were identified using 2D-DIGE. To our knowledge, this is the first time this technique is applied to study bacterial glycoproteomics. The structure of the glycan used to decorate these proteins in *A. baumannii* was determined by a combination of MS and NMR techniques. The sugar was determined to be a pentasaccharide of the formula β-GlcNAc3NAcA4OAc-4-(β-GlcNAc-6-)-α-Gal-6-β-Glc-3-β-GalNAc-S/T (Fig S4). The glycan contains a terminal *O*-acetylated glucuronic acid derivative that is negatively charged and has not previously been described. A similar monosaccharide was found in *Pseudomonas aeruginosa* and *Bordetella pertussis* [45]. Of the glycoproteins identified, only one (A1S_1193; MotB) has any significant homology outside of the genus *Acinetobacter*, with the remaining being annotated as hypothetical proteins. MotB has homology with proteins such as Pal from *Haemophilus influenzae* that have been shown to bind to peptidoglycan and stabilize the outer membrane [46]. Functional characterization of *A. baumannii* glycoproteins will be crucial to explain the phenotypes associated with lack of glycosylation.

Biofilms are proposed to be a virulence factor that is associated with increased antibiotic resistance, pathogenicity, and persistence of a bacterial population [47]–[49]. We have found that *O*-glycosylation enhances biofilm formation by *A. baumannii* ATCC 17978. Biofilm formation is a multistep process that involves an initial weak association leading to an irreversible attachment, which leads eventually to a complex maturation into sophisticated superstructures [50]. We observed by flow cell and confocal imaging that glycosylation enhances the initial attachment as well as mature biofilm mass and density. It is tempting to speculate that glycans of the glycoproteins may have a function in cell-to-cell adhesion [51]. Further work will elucidate in which aspect protein glycosylation is required for efficient biofilm formation.

The basic mechanisms of phagocytic cells are used in both amoebae and mammalian macrophages. As an infection model, the amoebae *D. discoideum* is considered a primitive macrophage. *D. discoideum* cells were unable to predate on *A. baumannii* WT lawns, but were able to efficiently predate on lawns of the glycosylation-deficient bacteria. It is uncertain how protein *O-*glycosylation protects *A. baumannii* from *D. discoideum* but we can hypothesize that glycosylation may help in the inhibition of phagocytosis by the amoebae, and/or prevent bacterial lysis by reactive oxygen species produced by the amoebae [52]. Another possibility is that glycosylation of certain proteins is required to interfere with bacterial degradation and intracellular vesicle transport and/or fusion, as shown for *Legionella* [53]. We also analyzed if protein *O-*glycosylation plays a role in pathogenesis in *G. mellonella* caterpillars. This model system has been recently shown to recreate the mammalian humoral immune system, with similar antimicrobial peptides, toll-like receptors, and the complement-like mechanism of melanization [54]. Similar to the *D. discoideum* model, *A. baumannii* Δ*pglL* strain was unable to kill *G. mellonella*. *O-*glycosylation could mediate killing of the larvae by stabilizing the bacterial outer membrane of *A. baumannii*, which could prevent killing by antimicrobial peptides. The negative charges of the glycan chains could play a role in this process. Alternatively, glycosylation could mask signals detected by the larvae or prevent phagocytosis by *G. mellonella* haemocytes, among other possibilities. The involvement of glycoproteins in virulence is further supported by the demonstration that the *ΔpglL* strain is outcompeted by wild type bacteria in a murine septicemia model. Thus, our experiments showed that glycosylation is critical for virulence in three different model systems. Further work using strains carrying mutations in individual glycoproteins will help to elucidate the exact role of protein glycosylation in pathogenesis.

Glycoproteins are usually immunodominant in bacteria, and therefore, the glycoproteins identified in this study may be the base of future vaccine formulations and diagnostic methods. The prevalence of the *O*-glycosylation machinery in *Acinetobacter* sp., together with its role in virulence in the three different pathogenesis models, suggest that protein *O*-glycosylation represents a novel target for the development of antibiotics that could be key to prevent further dissemination of this emerging human pathogen, which has become a major threat to our healthcare systems.

## Materials and Methods

### Bacterial strains, plasmids, growth conditions, and antimicrobial agents

The bacterial strains and plasmids used in this study are listed in Table 3. *A. baumannii* strains were grown in Luria Bertani broth/agar at 37°C. The antibiotics ampicillin (Ap) 100 µg/mL, gentamicin (Gm) 50 µg/mL, and tetracycline (Tc) 5 µg/mL were added for selection as needed.

**Table 3:** List of strains, plasmids, and primers used in this work.

Strains	Reference
*A. baumannii* ATCC 17978	[68]
*A. baumannii* ATCC 17978 Δ*pglL*	This work

Plasmid	Reference
pEXT20	[69]
pWH1266	[70]
pFLP2	[71]
pSPG1	[55]

Primers	Sequence (5′-3′)
*pglL*_fwd_ EcoRI	gcgaattcatgttttctagtgaaatgggtgcctttgcagcag
*pglL*_rev_ XbaI 10His	gaggaattacattcggctttggatacaccaccaccaccaccaccaccaccaccactaatctagaaatt
*aacC1*_int rev_	catcgcgcttgctgccttcgaccaagaagc
K/O *pglL*_fwd_ EcoRI	gagaattcatgaatgcacaaaaaggttttacattaattgaactcatg
K/O *pglL_rev_* XbaI	ccgagttgttcaattccccaatcattaaacatacgagtctagatgt

### Construction of *A. baumannii* Δ*pglL* knockout and *in trans* complementation

In order to create a Δ*pglL* via homologous recombination, we cloned a ∼3500 bp fragment consisting of ∼1000 bp upstream and downstream of A1S_3176 into pEXT20 using primers K/O *pglL*_fwd_ and K/O *pglL*_rev_ from *A. baumannii* ATCC 17978 genomic DNA (Table 3). The construct was subsequently subcloned from pEXT20 into pFLP2. We then digested pFLP2-*pglL* with PsiI and replaced A1S_3176 with a SmaI excised Gentamicin resistance cassette (*aacC*1) from pSPG1 [55]. The plasmid pFLP2 does not replicate in *A. baumannii* ATCC 17978. This final construct was transformed into electro-competent *A. baumannii* WT cells and selection for a single recombination event was analyzed using media supplemented with gentamicin. Positive colonies were grown in 5 mL LB at 37°C for 72 hours, with 1/1000 re-inoculations into fresh LB media every 24 hour period. After 72 hours, the liquid culture was plated on LB agar supplemented with gentamicin and 10% sucrose to select for a double recombination event. Colony PCR using both internal and external primers showed the allelic exchange of A1S_3176 with *aacC1*, generating a knockout mutant of *A. baumannii pglL*.

### SDS-PAGE and Periodic acid stain (PAS) analysis of membrane extracts

Bacterial cultures were pelleted by centrifugation for 15 mins at 10,000×g, washed with PBS, resuspended in PBS, and subsequently lysed by French Press. Unbroken cells were pelleted by centrifugation for 15 mins @ 5,000×g. The supernatant was ultracentrifugated for 1 hr @ 100,000×g (4°C) to pellet cell membrane. Samples were quantified by Bradford protein quantification (Biorad) and analyzed on a 12% SDS-PAGE. The PAS stain protocol used was previously described [56].

### LPS extraction protocol

LPS was extracted according to Marolda *et al* [57]. Samples were resuspended in 50 µL of dH_2_0 and analyzed by Silverstain on a 15% SDS-PAGE.

### 2D-DIGE analysis of *A. baumannii* membrane extracts

Lipid-free membranes were obtained for 2D-DIGE analysis according to [27]. The material was resuspended in: 6.5 M Urea, 2.2 M thiourea, 1% w/v ASB-14, 5 mM Tris-HCl pH 8.8, 20 mM DDT, 0.5% IPG buffer. The samples were labeled using CyDye minimal labeling protocol (Amersham Biosciences). *A. baumannii* WT membranes were labeled with Cy5 and Δ*pglL* were labeled with Cy3. Samples were quantified by 2D-Quant kit (GE Healthcare) and 600 µg of each WT and Δ*pglL* membranes were mixed in Destreak solution (GE Healthcare) to a final volume of 450 µL. 24 cm pH 3–11 NL IPG strips were simultaneously rehydrated and sample loaded for 24 hrs at room temperature in the dark. Isoelectric focusing was done using the Ettan IPGphor system for a total of 56,000 Vhr in the dark. The strip was then incubated in 10 mL of equilibration solution (2% SDS, 50 mM Tris-HCl, 6 M Urea, 30% (v/v) glycerol, 0.002% bromophenol blue) for 15 mins with 100 mg DTT and then 10 mL equilibration solution with 250 mg iodoacetamide. The strip was then sealed into a DALT 12.5 precast gel with 0.5% agarose. The system was run at 2.5 W/gel for 30 mins, the 17 W/gel until the dye front exited the bottom. The gel was visualized using FLA-5000 (FujiFilm) and the images analyzed by ImageQuant 5.0. The gel was subsequently stained with Coomassie brilliant blue, and individual spots excised and prepared for mass spectrometry.

### MALDI-TOF/TOF MS and MS/MS analysis of glycoproteins

Samples were in gel tryptically-digested and the peptides were desalted using C_18_ Zip-Tips and eluted with 60% CH_3_CN/40% H_2_O. Samples were spotted on a Bruker Daltonics MTP ground steel or Bruker Daltonics MTP AC600 Anchorchip target plate and air dried. 1 µL for ground steel and 0.4 µL for the AC600 target of 2,5-dihydroxybenzoic acid (DHB, 10 mg/mL in 30% H_2_O, 70% CH_3_CN) was spotted on top and allowed to dry. Mass spectra were obtained in the positive mode of ionization using a Bruker Daltonics (Bremen, GmbH) UltrafleXtreme MALDI TOF/TOF mass spectrometer. The FlexAnalysis software provided by the manufacturer was used for analysis of the mass spectra.The MS/MS spectra were obtained manually. The exact m/z used as the precursor m/z for MS/MS was determined first on a Bruker Daltonics (Billerica, MA) Apex Qe MALDI FTICR MS instrument and the MS/MS spectrum was automatically re-calibrated based upon this m/z.

### MALDI TOF-TOF MS characterization of the *A. baumannii O*-glycan from membrane extracts

Lipid free membrane extracts were digested for 72 hrs at 37°C with 2 µL Pronase E (20 mg/mL) being freshly added every 24 hrs. Glycosylated peptides were enriched using Active Charcoal Micro SpinColumn (HARVARD Apparatus) Briefly, the column was prewashed 3× with 400 µL of 0.1% TFA in of 80% ACN and 20% ddH2O and centrifuged at 500 RCF for 2 minutes. The column was equilibrated 3× using 400 µL of H_2_O. The sample was loaded 3× at 500 RCF for 2 minutes. The column was washed 2× with 200 µL of ddH_2_O at 500 RCF for 2 minutes. The glycan was eluted 3× with 100 µL 0.1% TFA in 50% ACN and 50% H2O at 1000 RCF for 2 minutes. The sample was dried by vacuum centrifugation and analyzed by MALDI-TOF/TOF MS and MS/MS.

### Purification of glycans for NMR analysis

For NMR analysis glycoproteins were digested with a large excess of proteinase K at pH 8 (adjusted by addition of ammonia) at 37°C for 48 hours. Products of digestion or free oligosaccharides were separated on Sephadex G-15 column (1.5×60 cm) and each fraction eluted before salt peak was dried and analyzed by ^1^H NMR. Fractions containing desired products were separated by anion exchange chromatography on Hitrap Q column (5 mL size, Amersham) and glycan eluted with a linear gradient of NaCl (0–1 M, 1 h). Desalting was performed on Sephadex G15 prior to analysis by NMR.

### NMR spectroscopy analysis

NMR experiments were carried out on a Varian INOVA 600 MHz (^1^H) spectrometer with 3 mm gradient probe at 25°C with acetone internal reference (2.225 ppm for ^1^H and 31.45 ppm for ^13^C) using standard pulse sequences DQCOSY, TOCSY (mixing time 120 ms), ROESY (mixing time 500 ms), HSQC and HMBC (100 ms long range transfer delay). AQ time was kept at 0.8–1 sec for H-H correlations and 0.25 sec for HSQC, 256 increments was acquired for t1. Assignment of spectra was performed using Topspin 2 (Bruker Biospin) program for spectra visualization and overlap. Monosaccharides were identified by COSY, TOCSY and NOESY cross peak patterns and ^13^C NMR chemical shifts. Aminogroup location was concluded from high field signal position of aminated carbons (CH at 45–60 ppm). Connections between monosaccharides were determined from transglycosidic NOE and HMBC correlations.

### Protease digestion and enrichment of glycopeptides by zwitterionic hydrophilic interaction chromatography (ZIC-HILIC)

Dried membrane protein-enriched fractions were resuspended in 6 M urea, 2 M thiourea, 40 mM NH_4_HCO_3_. Samples were reduced, alkylated, digested with Lys-C (1/200 w/w) and then trypsin (1/50 w/w) as described previously [20]. Digested samples were then dialyzed against ultra-pure water overnight using a Mini Dialysis Kit with a molecular mass cut off of 1000 Da (Amersham Biosciences, Buckinghamshire, UK) and on completion were collected and lyophilized. ZIC-HILIC enrichment was performed according to [20] with minor modifications. Micro-columns composed of 10 µm ZIC-HILIC resin (Sequant, Umeå, Sweden) were packed into P10 tips on a stage of Empire C_8_ material (Sigma) to a bed length of 0.5 cm and washed with ultra-pure water prior to use. Dried digested samples were resuspended in 80% acetonitrile (ACN), 5% formic acid (FA) and insoluble material removed by centrifugation at 20,000×*g* for 5 min at 4°C. Samples were adjusted to a concentration of 2 µg/µL and 100 µg of peptide material loaded onto a column and washed with 10 load volumes of 80% ACN, 5% FA. Peptides were eluted with 3 load volumes of ultra-pure water into low-bind tubes and concentrated using vacuum centrifugation.

### Identification of glycopeptides using reversed phase LC-MS and HCD MS-MS

ZIC-HILIC fractions were resuspended in 0.1% FA and loaded onto a Acclaim PepMap 100 µm C18 Nano-Trap Column (Dionex Corporation, Sunnyvale, CA) for 10 min using a UltiMate 3000 intelligent LC system (Dionex Corporation). Peptides were eluted and separated on 20 cm, 100 µm inner diameter, 360 µm outer diameter, ReproSil – Pur C_18_ AQ 3 µm (Dr. Maisch, Ammerbuch-Entringen, Germany) in house packed column. Enriched peptides derived from tryptic digests were analysed using an LTQ-Orbitrap Velos (Thermo Scientific, San Jose CA). Samples were eluted using a gradient from 100% buffer A (0.5% acetic acid) to 40% buffer B (0.5% acetic acid, 80% MeCN) over 120 mins at a constant flow of 200 nL/min enabling the infusion of sample in the instrument using ESI. The LTQ-Orbitrap Velos was operated using Xcalibur v2.2 (Thermo Scientific) with a capillary temperature of 200°C in a data-dependent mode automatically switching between MS ion trap CID and HCD MS-MS. For each MS scan, the three most abundant precursor ions were selected for fragmentation with CID, activation time 30 ms and normalized collision energy 35, followed by HCD, activation time 30 ms and normalized collision energy 45. MS resolution was set to 60,000 with an ACG of 1e^6^, maximum fill time of 500 ms and a mass window of *m/z* 600 to 2000. MS-MS fragmentation was carried out with an ACG of 3e^4^/2e^5^ for CID/HCD and maximum fill time of 100 ms/500 ms CID/HCD. For HCD events an MS resolution of 7500 was set. A total of six HILIC enrichments were performed and analysis by the above protocol.

### Database interrogation of identified glycopeptides

Raw files were processed within Proteome Discover version 1.0 Build 43 (Thermo Scientific) to generate .mgf files. To identify possible glycopeptides within exported scans, the MS-MS module of GPMAW 8.2 called ‘mgf graph’ was utilized. This module allowed the identification of all scan events within the generated .mgf files containing the diagnostic oxonium *m/z* 301.10 ion. These scan events were manually inspected and identified as possible glycopeptides based on the presence of the deglycosylated peptide ion with a tolerance of 20 ppm. To facilitate glycopeptide assignments from HCD scan events, ions below the mass of the predicted deglycosylated peptides were extracted with Xcalibur v2.2 using the Spectrum list function. Ions with a deconvoluted mass above the deglycosylated peptide mass and ions corresponding to known carbohydrate oxonium ions such as 204.08 and 366.14 were removed in a similar approach to post-spectral processing of ETD data [58], [59]. MASCOT v2.2 searches were conducted via the Australasian Proteomics Computational Facility (www.apcf.edu.au) with the Proteobacteria taxonomy selected. Searches were carried out with a parent ion mass accuracy of 20 ppm and a product ion accuracy of 0.02 Da with no protease specificity, instrument selected as MALDI-QIT-TOF (use of this instrumentation setting was due to the observation of multiple internal cleavage products, extensive NH_3_ and H_2_O loss from *a*, *b*, *y* ions, which are all included within this scoring setting) as well as the fixed modification carbamidomethyl (C) and variable modifications, oxidation (M) and deamidation (N). An ion score cut-off of 20 was accepted and all data were searched with the decoy setting activated generating a zero false positive rate generated against the decoy database.

### Biofilm analysis using 96 well polystyrene plates

Cultures were grown overnight and re-inoculated at an OD_600_ 0.05 in 100 µL into replicates in a 96 well polystyrene plate (Costar). The cultures were subsequently grown without shaking for 48 hours at 30°C. Bacterial growth was determined by measuring the absorbance at OD_600 nm_. The cultures were removed and the wells washed with ddH_2_0, followed by the addition of 100 µL of 1% crystal violet in ethanol to stain the cells. The plate was incubated for 30 mins with gentle agitation, then thoroughly washed with ddH_2_0, and the stained biofilms solubilized with 100 µL of 2% SDS for 30 minutes with gentle agitation. The amount of biofilm formed was quantified by measuring the absorbance at OD_580 nm_. The data was normalized using the ratio between OD_580_/OD_600_.

### Flow cell biofilm experiments, fluorescent staining and confocal laser scanning microscopy

Flow cell experiments and fluorescent staining were performed as described previously by Seper et al. [60]. Briefly, the respective overnight cultures were adjusted to OD_600_ = 0.1 using 50-fold diluted LB (2%). Per channel, approximately 250 µl of the dilutions were inoculated. After static incubation for 2 h, flow of pre-warmed 2% LB (37°C) was initiated (3 ml/h). Biofilms were allowed to form for a time period of 24 h and were stained with SYTO 9 (Invitrogen) for visualization. Images of attached bacteria or biofilms were acquired using a Leica SP5 confocal microscope (Leica Microsystems, Mannheim, Germany) with spectral detection and a Leica HCX PL APO CS 40× oil immersion objective (NA 1.25). For the SYTO 9 signal, the excitation wavelength was set at 488 nm and fluorescence emission was detected between 500–530 nm. Optical sections were recorded in 0.2 µm steps. For two-dimensional image visualization the Leica LAF and for three-dimensional image processing the AMIRA software (direct volume rendering with VOLREN module) was used. Quantification of image stacks was performed using COMSTAT (http://www.comstat.dk) [61] (M. Vorregaard et al., pers. comm.). For COMSTAT analysis at least six image stacks from three independent experiments were used.

### *Dictyostelium discoideum* virulence assay

This assay was performed essentially as described by [62]. Briefly, midlogarithmic cultures of *D. discoideum* were mixed with overnight cultures of bacteria to a final concentration of 1×10^3^ amoebae ml^−1^. 0.2 ml of the suspension was then plated on SM/5 agar containing 1% ethanol. Plates were incubated at room temperature and monitored for *D. discoideum* plaques for 3–5 days. Wild type bacteria are toxic to the amoebae. Appearance of plaques indicates attenuation.

### *Galleria mellonella* virulence assay

This assay was performed as previously described [32]. *Galleria mellonella* larvae were bred in sterile conditions at 37°C by Dr. Andrew Kedde (University of Alberta). After injection of bacteria, caterpillars were incubated at 37°C, and the number of dead caterpillars was scored every 5 hours. Caterpillars were considered dead when they were nonresponsive to touch. This experiment is a representative of 3 biological replicates.

### Murine bacterial competition model assay

A murine model of disseminated sepsis using BALB/c mice (16–20 grams) was used for bacterial challenge [63], [64]. *A. baumannii* strains were grown for 18 h at 37°C in Luria broth with appropriate antibiotics and adjusted to the appropriate concentration in physiologic saline. Inoculums were prepared by mixing the bacterial suspensions 1∶1 (v∶v) with a 10% solution (w/v) of porcine mucin (Sigma, St. Louis, MO) which increases the infectivity of *A. baumannii*, allowing for a lower concentration of bacteria to be used [65]–[67]. Mice were injected intraperitoneally with 0.2 ml of the bacterial/mucin inoculums. Bacterial concentrations were determined by plating dilutions on Luria agar. The wild type strain lethal dose for 50% of animals was determined by the limit test where groups of 5 mice were infected with dilutions of bacteria, at a range of concentrations within 2 logs of a concentration of bacteria that had previously been shown to be lethal with this species of bacteria using a disseminated sepsis model.

An *in vivo* competition assay was used to compare fitness between the wt and Δ*pglL* strains [37]–[39]. Liquid cultures containing individual strains were diluted and plated on LB agar. Mixed inoculums were established by mixing equal proportions of strains based on the OD_600_. Once mixed the inoculums were serially diluted and plated on LB agar and LB agar with gentamycin to select for the Δ*pglL*. The expected ratio of CFU on LB compared to CFU on LB with gentamycin was 2∶1.

For bacterial competition experiments *in vivo* an animal model of sepsis was used. Groups of 3 BALB/c female 16–20-g mice were inoculated intraperitoneally with 1×10^5^ CFUs of mixed inoculums (50% of each strain). Groups of 3 mice were sacrificed at 18 h after inoculation. Mice at 18 hours of infection were showing clinical signs of illness and were often moribund. Spleens were aseptically removed, weighed, and homogenized via passage through a cell strainer (BD falcon 70 um cell strainer) in physiological saline before plating serial log dilutions on Luria agar plates for bacterial quantification. If the two strains had equal fitness in vivo the ratio established prior to infection should be maintained.

### Ethics statement

All procedures and experiments involving animals (mice) were approved by the Institutional Animal Care Committee of Defence Research and Development Canada Suffield (protocol # CWS-08-1-1-1), and were in accordance with guidelines from the Canadian Council of Animal Care.

## Supporting Information

Figure S1 **Analysis of LPS extraction of** ***A. baumannii*** **strains resolved by SDS-PAGE and visualized by Silverstain.** Samples were as follows: lane 1 WT; lane 2 Δ*pglL*, lane 3 Δ*pglL*, pWH1266-*pglL*, 4-Δ*pglL*, pWH1266 control.
(TIF)

Figure S2 **Western Immunoblot of OmpA (A1S_2840) in whole cell extracts of** ***A. baumannii*** **ATCC 17978 strains.** Each lane was loaded with 0.2 OD600 of sample, and probed with OmpA monoclonal antibody with no observable differences between the WT and *ΔpglL*.
(TIF)

Figure S3 **MALDI-TOF/TOF MS/MS fingerprint analysis of glycosylated peptides in** ***A. baumannii*** **.** A) Sequencing of the peptide **SAGDQAASDIATATDNASAK** from the parental peak 2895.24 Da demonstrates the peptide matches the expected sequence of A1S_3626. B) Sequencing of the peptide **ETPKEEEQDKVETAVSEPQPQKPAK** from the parental peak 2822.33 Da demonstrates the peptide matches the expected sequence of A1S_3744.
(TIF)

Figure S4 **^1^H∶^13^C HSQC 2D NMR spectra of the** ***A. baumannii*** **O-glycan.** NMR data for the *A. baumannii O-*glycan (D_2_O, 28°C, 600 MHz). NAc: 1.95/23.0; 2.00/23.3; 2.04/23.5; 2.04/23.5 ppm, all C-1 at 175.9 ppm. OAc: 2.06/21.3, 174.0 ppm. The amino acids attached to the pentasaccharide were determined to be S-E-A (order not determined). * indicates impurity.
(TIF)

Figure S5 ***Dictyostelium discoideum*** **plaque assay comparing virulence of** ***A. baumannii*** **strains.** Bacteria were mixed with ∼500 amoebae, plated on SM/5 agar with 1% ethanol, and incubated at room temperature for 72 hours to allow for plaque formation. Results are representative of four independent experiments with 1 representing WT and 2 representing Δ*pglL*.
(TIF)

Figure S6 **Protein glycosylation appears to be highly conserved in** ***A. baumannii*** **clinical isolates.** A) 10 µg of membrane extract from *A. baumannii* clinical isolates obtained from the University of Alberta Hospital were resolved by SDS-PAGE and detection of carbohydrates was performed by PAS stain. 8/8 isolates have a similar PAS reactive band to *A. baumannii* ATCC 17978. Isolates were putatively identified by sequencing 16S rDNA and *recA* and are as follows: lane 1 *A. calcoaceticus*, lane 2 *A. calcoaceticus*, lane 3 *A. pittii*, lane 4 *A. pittii*, lane 5 *A. nosocomialis*, lane 6 *A. nosocomialis*, lane 7 *A. junii*, lane 8 *A. baumannii*. B) Phylogenetic tree of hypothetical *O-*OTases of *Acinetobacter* sp. and known *O-*OTases and *O-*antigen ligases. Protein identification numbers are as follows: *N. meningitidis* M58 PglL (NP_273640.1); *N. gonorrhoeae* MS11 PglO (ZP_04726765.1); *P. aeruginosa* PAO1 PilO (AAP43787.1); *A. baumannii* ATCC 17978 (YP_001086175.1); *A. baumannii* AB900 (ZP_04661261.1); *A. baumannii* ACICU (YP_001848035.1); *A. baumannii* ATCC 19606 (ZP_05829147.1), *A. baumannii* ATCC 19606 Ligase (YP_001713790.1); *A. baumannii* SDF (YP_001705998.1); *A. baumannii* 1656-2 (ADX05061.1); *A. baumannii* 6014059 (ZP_08441731.1); *A. calcoaceticus* RUH2202 (ZP_06059388.1); *A. baumannii* AYE (YP_001712289.1); *A. baumannii* AB0057 (YP_002320930.1); *A. calcoaceticus* PHEA-2 (ADY83231.1); *Acinetobacter* sp. SH024 (ZP_06693023.1); *Acinetobacter* sp. DR1 (YP_003730587.1); *Acinetobacter* sp. RUH2624 (ZP_05825054.1); *Acinetobacter* sp. ATCC_2724 (ZP_03824224.1); *A. junii* SH205 (ZP_06066893.1); *A. haemolyticus* ATCC 19194 (ZP_06729056.1); *Acinetobacter* sp. ADP1 ACIAD0103 (YP_044903.1); *Acinetobacter* sp. ADP1 ACIAD3337 (YP_047828.1); *A. lwoffii* SH145 (ZP_06070298.1); *Klebsiella pneumoniae* WaaL (AAX20101.1). Phylogenetic tree was built using http://www.phylogeny.fr/version2_cgi/index.cgi (50).
(TIF)
