# Supplementary figures and images for: Identification of a General O-linked Protein Glycosylation System in Acinetobacter baumannii and Its Role in Virulence and Biofilm Formation
Source: PLoS Pathog. 2012 Jun 7;8(6):e1002758. doi: 10.1371/journal.ppat.1002758 (PMC3369928; doi:10.1371/journal.ppat.1002758)

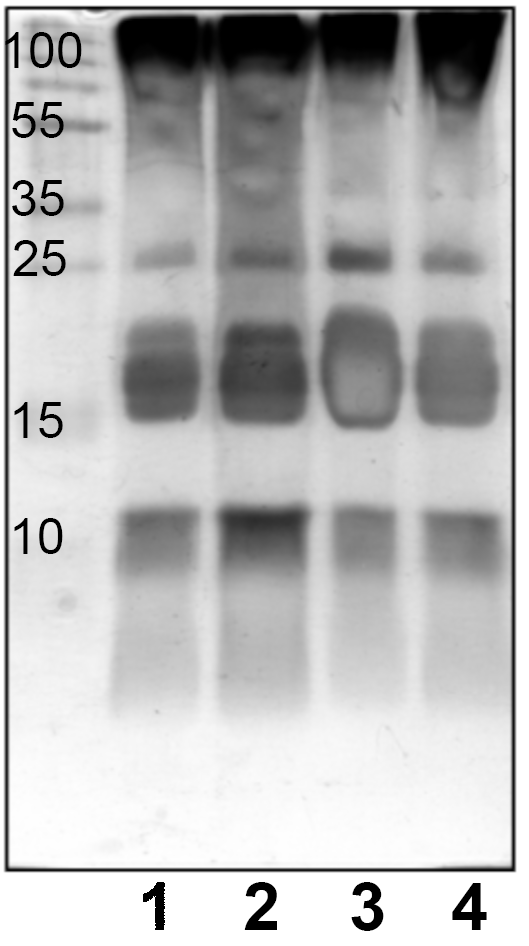

Supplement: Figure S1 — Analysis of LPS extraction of A. baumannii strains resolved by SDS-PAGE and visualized by Silverstain. Samples were as follows: lane 1 WT; lane 2 ΔpglL, lane 3 ΔpglL, pWH1266-pglL, 4-ΔpglL, pWH1266 control. (TIF) [file ppat.1002758.s001.tif]

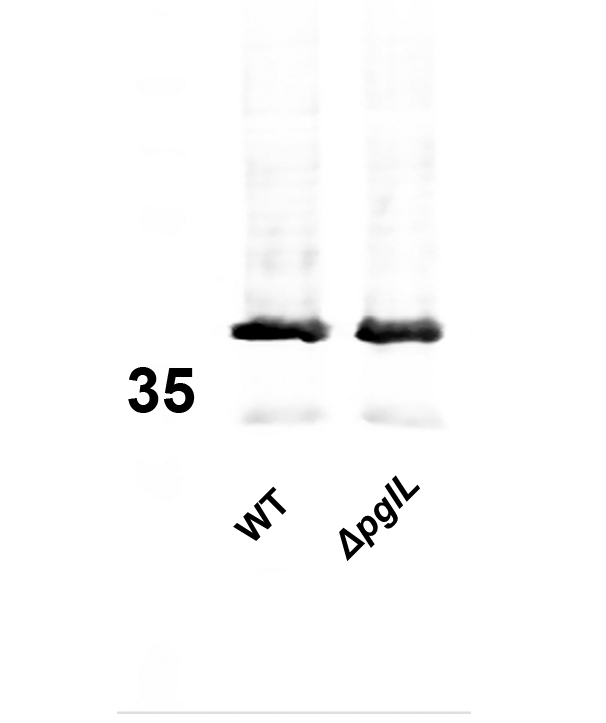

Supplement: Figure S2 — Western Immunoblot of OmpA (A1S_2840) in whole cell extracts of A. baumannii ATCC 17978 strains. Each lane was loaded with 0.2 OD600 of sample, and probed with OmpA monoclonal antibody with no observable differences between the WT and ΔpglL. (TIF) [file ppat.1002758.s002.tif]

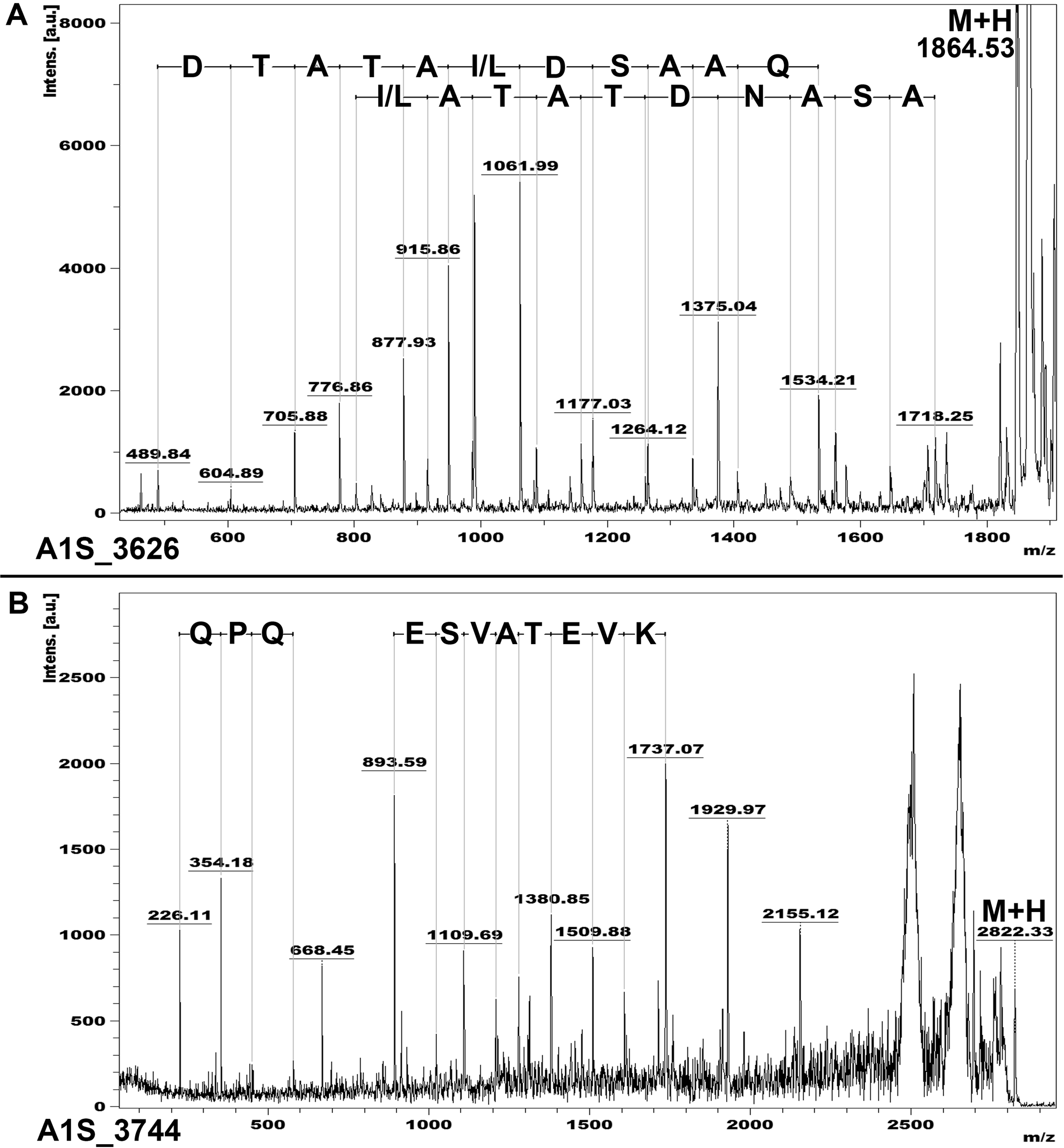

Supplement: Figure S3 — MALDI-TOF/TOF MS/MS fingerprint analysis of glycosylated peptides in A. baumannii . A) Sequencing of the peptide SAGDQAASDIATATDNASAK from the parental peak 2895.24 Da demonstrates the peptide matches the expected sequence of A1S_3626. B) Sequencing of the peptide ETPKEEEQDKVETAVSEPQPQKPAK from the parental peak 2822.33 Da demonstrates the peptide matches the expected sequence of A1S_3744. (TIF) [file ppat.1002758.s003.tif]

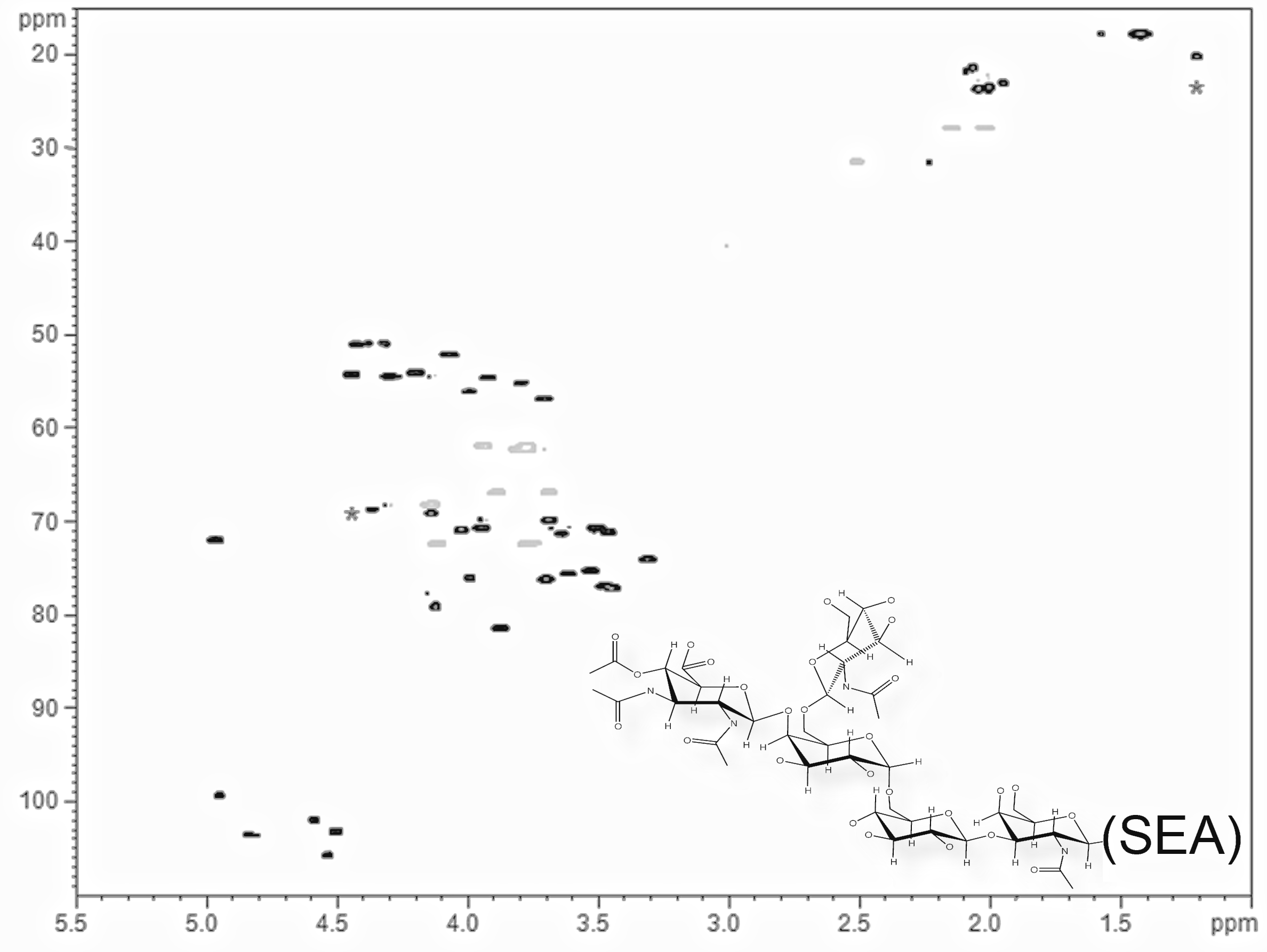

Supplement: Figure S4 — 1H∶13C HSQC 2D NMR spectra of the A. baumannii O-glycan. NMR data for the A. baumannii O-glycan (D2O, 28°C, 600 MHz). NAc: 1.95/23.0; 2.00/23.3; 2.04/23.5; 2.04/23.5 ppm, all C-1 at 175.9 ppm. OAc: 2.06/21.3, 174.0 ppm. The amino acids attached to the pentasaccharide were determined to be S-E-A (order not determined). * indicates impurity. (TIF) [file ppat.1002758.s004.tif]

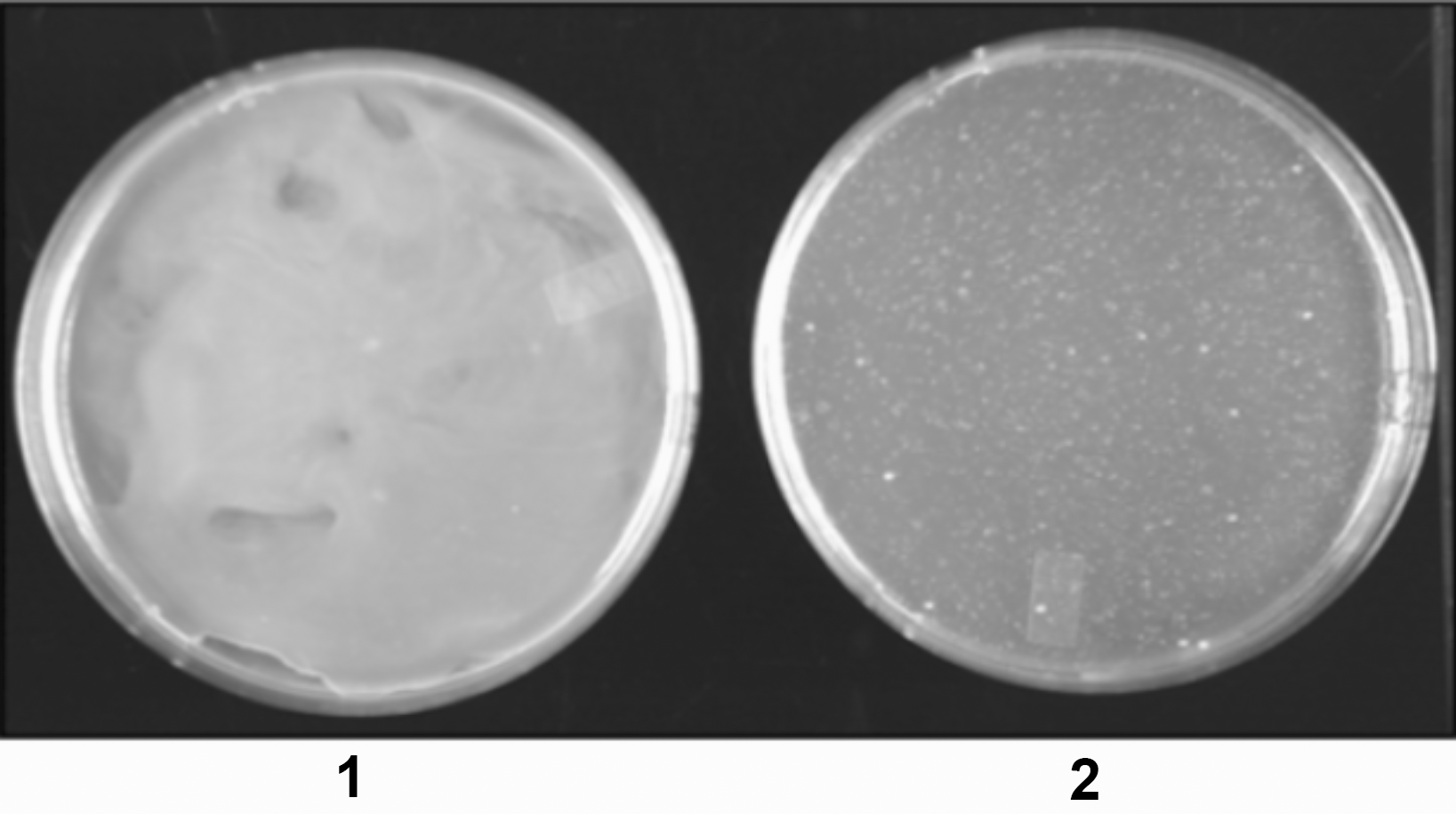

Supplement: Figure S5 — Dictyostelium discoideum plaque assay comparing virulence of A. baumannii strains. Bacteria were mixed with ∼500 amoebae, plated on SM/5 agar with 1% ethanol, and incubated at room temperature for 72 hours to allow for plaque formation. Results are representative of four independent experiments with 1 representing WT and 2 representing ΔpglL. (TIF) [file ppat.1002758.s005.tif]

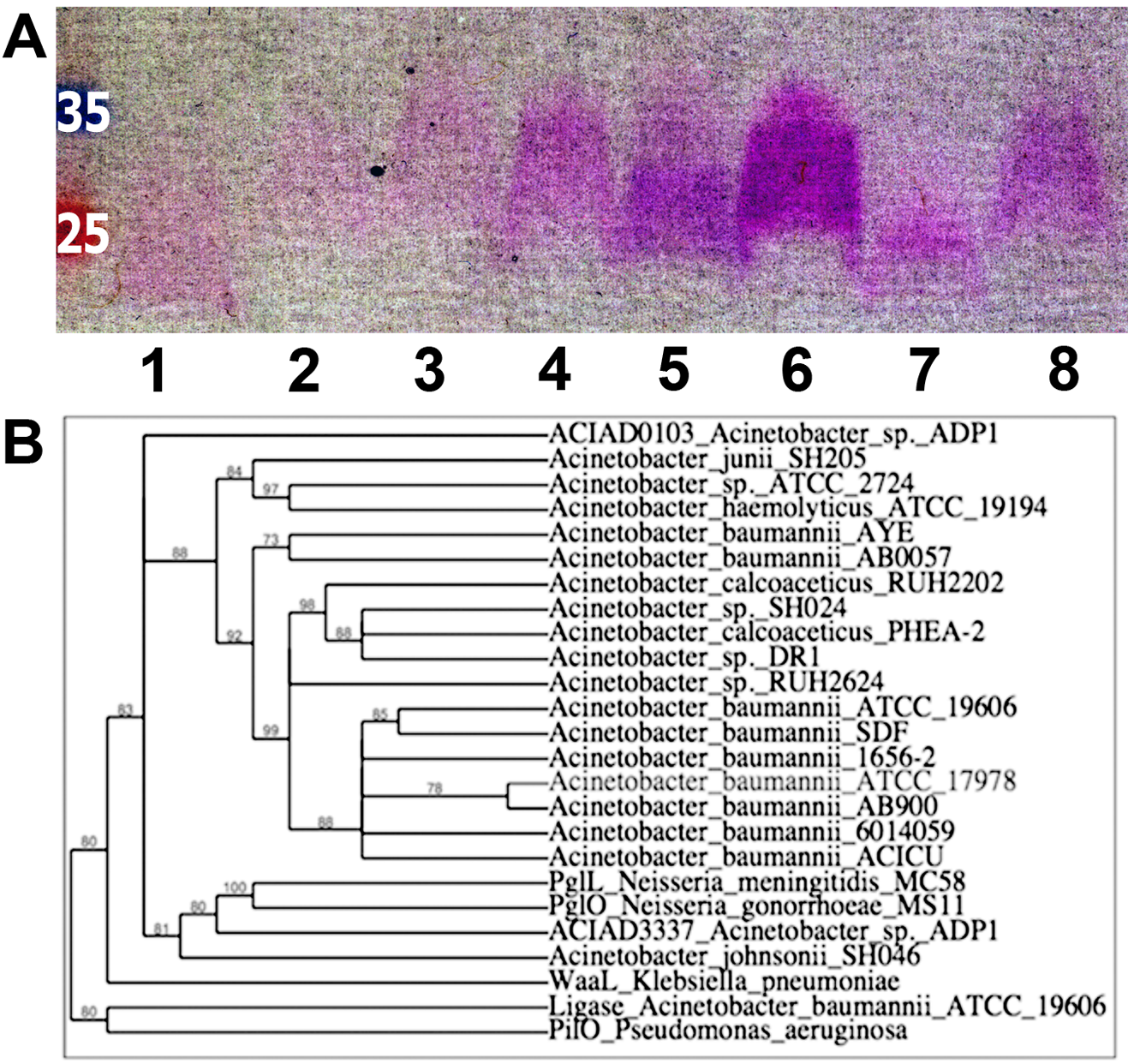

Supplement: Figure S6 — Protein glycosylation appears to be highly conserved in A. baumannii clinical isolates. A) 10 µg of membrane extract from A. baumannii clinical isolates obtained from the University of Alberta Hospital were resolved by SDS-PAGE and detection of carbohydrates was performed by PAS stain. 8/8 isolates have a similar PAS reactive band to A. baumannii ATCC 17978. Isolates were putatively identified by sequencing 16S rDNA and recA and are as follows: lane 1 A. calcoaceticus, lane 2 A. calcoaceticus, lane 3 A. pittii, lane 4 A. pittii, lane 5 A. nosocomialis, lane 6 A. nosocomialis, lane 7 A. junii, lane 8 A. baumannii. B) Phylogenetic tree of hypothetical O-OTases of Acinetobacter sp. and known O-OTases and O-antigen ligases. Protein identification numbers are as follows: N. meningitidis M58 PglL (NP_273640.1); N. gonorrhoeae MS11 PglO (ZP_04726765.1); P. aeruginosa PAO1 PilO (AAP43787.1); A. baumannii ATCC 17978 (YP_001086175.1); A. baumannii AB900 (ZP_04661261.1); A. baumannii ACICU (YP_001848035.1); A. baumannii ATCC 19606 (ZP_05829147.1), A. baumannii ATCC 19606 Ligase (YP_001713790.1); A. baumannii SDF (YP_001705998.1); A. baumannii 1656-2 (ADX05061.1); A. baumannii 6014059 (ZP_08441731.1); A. calcoaceticus RUH2202 (ZP_06059388.1); A. baumannii AYE (YP_001712289.1); A. baumannii AB0057 (YP_002320930.1); A. calcoaceticus PHEA-2 (ADY83231.1); Acinetobacter sp. SH024 (ZP_06693023.1); Acinetobacter sp. DR1 (YP_003730587.1); Acinetobacter sp. RUH2624 (ZP_05825054.1); Acinetobacter sp. ATCC_2724 (ZP_03824224.1); A. junii SH205 (ZP_06066893.1); A. haemolyticus ATCC 19194 (ZP_06729056.1); Acinetobacter sp. ADP1 ACIAD0103 (YP_044903.1); Acinetobacter sp. ADP1 ACIAD3337 (YP_047828.1); A. lwoffii SH145 (ZP_06070298.1); Klebsiella pneumoniae WaaL (AAX20101.1). Phylogenetic tree was built using http://www.phylogeny.fr/version2_cgi/index.cgi (50). (TIF) [file ppat.1002758.s006.tif]
